# Individual-level movement bias leads to the formation of higher-order social structure in a mobile group of baboons

**DOI:** 10.1098/rsos.170148

**Published:** 2017-07-12

**Authors:** Tyler R. Bonnell, Parry M. Clarke, S. Peter Henzi, Louise Barrett

**Affiliations:** 1 Department of Psychology, University of Lethbridge, Alberta, Canada; 2 Applied Behavioural Ecology and Ecosystems Research Unit, University of South Africa, Florida, South Africa

**Keywords:** movement ecology, leadership, network core/periphery, attraction–repulsion models

## Abstract

In mobile social groups, influence patterns driving group movement can vary between democratic and despotic. The arrival at any single pattern of influence is thought to be underpinned by both environmental factors and group composition. To identify the specific patterns of influence driving travel decision-making in a chacma baboon troop, we used spatially explicit data to extract patterns of individual movement bias. We scaled these estimates of individual-level bias to the level of the group by constructing an influence network and assessing its emergent structural properties. Our results indicate that there is heterogeneity in movement bias: individual animals respond consistently to particular group members, and higher-ranking animals are more likely to influence the movement of others. This heterogeneity resulted in a group-level network structure that consisted of a single core and two outer shells. Here, the presence of a core suggests that a set of highly interdependent animals drove routine group movements. These results suggest that heterogeneity at the individual level can lead to group-level influence structures, and that movement patterns in mobile social groups can add to the exploration of both how these structures develop (i.e. mechanistic aspects) and what consequences they have for individual- and group-level outcomes (i.e. functional aspects).

## 1. Introduction

Collective behaviour has been well documented in social insect aggregations, fish schools and bird flocks [1], and the constituent studies have done much to identify the nature of individual-level decisions, as well as explain how these underpin the emergence of adaptive group-level outcomes [2]. Less, however, is known about the drivers of collective action in stable social groups, such as those characteristic of diurnal primates, where repeated encounters occur among long-lived social partners [3,4]. Animals in such groups have the potential to develop consistent patterns of movement that are regulated—directly or indirectly—by the actions of others [5,6].

Recent studies of decision-making in moving baboon troops (*Papio hamadryas* spp*.*) have begun to address these questions directly. Strandburg-Peshkin *et al*. [3] found that individual members of an olive baboon group (*P. h. anubis*) adjusted their movement to conform to that of the numerically larger fraction when competing factions were attempting to initiate group movement. On the face of it, this suggests the absence of, or at least a limited role for, the influence of specific dyadic relationships within the group during the initiation of movement. In a subsequent study, however, Farine *et al*. [7] found that the locations of common spatial associates provided the best estimate of an individual's future location after 10 min of movement. Similarly, a study of chacma baboons (*P. h. ursinus*) found that animals preferentially followed close social associates when leaving sleeping sites [4,8]. Taken together, these studies suggest that social relations serve to regulate patterns of collective movement, but that individual influence may vary with context. Alternatively, such patterns may reflect the confounding of individual influence with group-level processes. That is, individuals with a large number of associates may inevitably be members of a larger competing faction in the group, such that individuals' tendencies to follow specific associates is subsumed within the formation of larger, more socially influential, subgroups. More generally, our suggestion is that individual influence may give rise to higher-order group structures that can explain patterns of collective movement. That is, while Strandburg-Peshkin *et al*. [3] and Farine *et al*. [7] are concerned with how animals reach consensus in collective decision-making, and how these influence the emergent structure of the group-level properties in terms of its speed, shape and directedness [9], we are interested in exploring whether individual movements can identify emergent social structures within the group.

Here, we examine individual decision-making by adults in a chacma baboon group while on the move (that is, travelling and/or foraging) [10,11] to assess whether individual-level processes can be linked to larger-scale group-level structures in just this fashion. Given evidence in other troops for both individual specific and averaged responses to local conditions, we first assess the degree to which animals' observed movement can be predicted both by the group as a whole and by the locations of specific animals. The estimates from this approach can identify whether there is consistent movement towards or away from particular animals in the group as well as the group centre, and can compare their relative weight in predicting observed movements. We interpret consistent movements towards/away from particular group members as social influence and characterize these as attractions and repulsions. We apply this approach from the point of view of each adult member of the group, providing a picture of the relative dependence of each individual, both on other group members' positions and the group as a whole during movement.

Second, in cases where there is evidence for consistent attractions and repulsions between pairs of animals, we make predictions about the direction of influence. Foraging experiments in chacma baboons suggest that sex and social rank are likely to influence the movement decisions of individuals [12]. We therefore predict that higher-ranking animals will have more influence on lower-ranked individuals' movement decision-making. We cannot fully assess the influence of sex due to a small sample size of males.

Finally, we evaluate the extent to which the influence between specific animals generates dependence structures at the level of the group [13]. Previous work at our study site provided evidence for distributed (i.e. democratic) decision-making [8], although the study was short and considered departures from sleeping sites only. In addition, experimental work on another chacma baboon population has suggested that decision-making is despotic and driven largely by one or a very few individuals [11]. Both options are therefore plausible, but given previous work on this same troop, we predict that influence patterns between specific animals will generate dependence structures which distribute influence widely across the adult network.

## 2. Material and methods

### 2.1. Data

Data were collected on the spatial locations of the 14 adult members of a baboon group (total group size, *N *≈ 45 individuals) at the De Hoop Nature Reserve [14] in South Africa on 74 full-day follows (approx. 12 h) between March 2007 and April 2008 [10,11]. All individuals were individually recognizable from natural markings and scars. A single observer (PMRC) moved back and forth through the group repeatedly, collecting a GPS point for all subjects encountered on each pass [11]. This resulted in a mean 9 min revisit time to each individual (median: 7 min) and generated 61 842 usable GPS points. We used ad hoc records of decided agonistic contest over the study period to assign each adult an ordinal dominance rank in a highly linear hierarchy. The Domicalc program [15] was used to confirm a high degree of linearity in the hierarchy (h1 = 1; *N* = 704; *p* < 0.0001). Adult group members are identified below by sex (M, F) and rank (1 = highest sex-specific rank).

### 2.2. Estimated individual influence: direction matching

**Figure 1.**
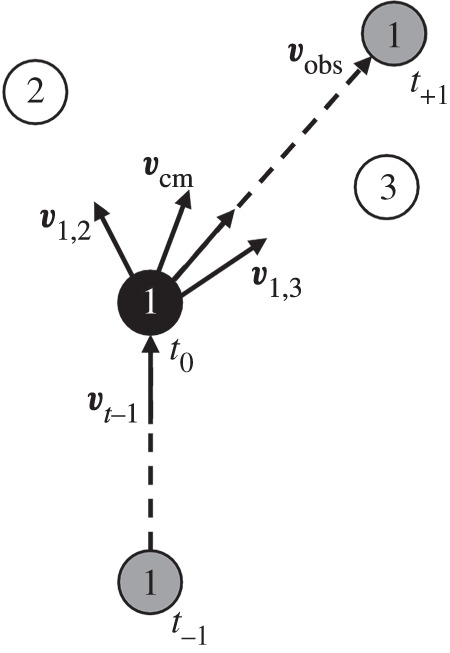
Visual diagram of equation (2.1) applied to an example group layout with three individuals. Equation (2.1) attempts to predict the direction of travel observed (${\hat{v}}_{\mathrm{obs}}$), taking into account the mean direction of the group from the focal individual (${\hat{v}}_{\mathrm{cm}}$), direction towards group members (e.g. ${\hat{v}}_{1,2}$) and the previous direction of travel (${\hat{v}}_{t-1}$).

We make use of our data to assess the extent to which the locations of group members consistently predict movement outcomes. From the point of view of a focal animal, we measured the direction of travel between two observations and predict its direction of travel using direction matching [16–18]. Specifically, the direction of travel at time *t* of each focal animal in turn was predicted on the basis of: (i) the direction of individual group members, (ii) the mean direction of the group as a whole, and (iii) its previous direction of travel, using the following equation (figure 1):

2.1 $${\hat{v}}_{t}=\overset{n}{\underset{i\neq j}{\sum }}\beta_{i,j}{\hat{v}}_{i,j}+\beta_{\mathrm{cm}}{\hat{v}}_{\mathrm{cm}}+\beta_{t-1}{\hat{v}}_{t-1},$$

where ${\hat{v}}_{t}$ is the predicted direction of travel and the ‘^’ notation indicates a unit vector describing direction of travel in two dimensions (*x*,*y*) at time *t*. The direction of travel at any time point *t* is predicted by: (i) ${\hat{v}}_{i,j}$, the direction to individual *j* from the focal animal *i*, allowing for the model to account for the location of each group member, (ii) ${\hat{v}}_{\mathrm{cm}}$, the mean direction from the focal animal to all other animals in the group, allowing the model to account for the group as a whole, and (iii) ${\hat{v}}_{t-1}$, the direction of travel from the previous observation, allowing the model to take into account the directional persistence of individual travel behaviour. The relative influence of each factor on movement behaviour is represented by *β* coefficients: previous heading (*β_t_*_−1_), mean group direction (*β*_cm_) and the influence of each group member *j* on the movement of individual *i* (*β_i_*_,*j*_). Given that the positions of individuals were not recorded concurrently, the positions of all other group members at any one given observation for a focal animal were estimated by linear interpolation between subsequent observations of group members. Using this approach, interpolation was used only to estimate the positions of other group members, whereas the positions of the focal animal were always based solely on our actual observations. The model was fitted within a Bayesian framework [19] with regularized priors (centred on 0 with a standard deviation of 0.1). Simulations of group movement patterns, degraded and interpolated to represent the temporal resolution and uncertainty in our observed data, were used to estimate the ability of equation (2.1) to identify influence patterns correctly, to aid in the interpretation of the results and to select for priors in equation (2.1) (electronic supplementary material, A). Given our treatment of *x* and *y* as independent, though they are not, we expect to have narrower standard errors around our estimates of movement bias. We therefore use 99% confidence intervals to estimate whether we have sufficient evidence for the correct sign of the effect (i.e. positive/negative movement bias). See the electronic supplementary material, B, for an alternative method that fully treats *x* and *y* as dependent, and results in qualitatively the same outcomes.

We also ran a second model to predict travel direction based on (i) previous direction of travel and (ii) mean direction from the focal animal to all other animals in the group (i.e. we removed all individual-specific influences). We compared this group-only model to the full model using WAIC [20]. This second model allows for an assessment of whether direction of travel is equally well predicted using only directional persistence and the group as a whole, or whether the location of individuals matters. It also helps determine whether the full model has overfit the data.

### 2.3. Influence patterns at the individual level

We tested the extent to which dominance rank could explain variation in influence patterns between individuals, using a linear-mixed model to quantify the variation in influence explained by differences in rank, controlling for the animal being influenced (*g_i_*):

2.2 $$\beta_{i,j}=\beta_{r}\Delta \text{ran}{\text{k}}_{i,j}+g_{i},$$

where *β_i_*_,*j*_ is the influence between individual *i* and individual *j*, Δrank*_i_*_,*j*_ is the difference in rank between individual *i* and *j*, *β*_r_ is the estimated effect of rank difference and *g_i_* is the random effect of the individual being influenced. Mixed models were fitted in R3.3.1 using the ‘rethinking’ package [21]. Normally distributed priors were used, centred on zero with a standard deviation of 0.1.

### 2.4. Influence patterns at the group level

We used social network analysis to visualize and interpret a group-level view of dependence among individuals. To construct the network, influence coefficients (*β_i_*_,*j*_) estimated from equation (2.1) were used as weighted edges. We removed links between individuals where the estimate of the coefficient contained 0 within its 99% credible intervals (CI). This network represents the extent to which each individual influences the movement decision-making of others. We calculated in- and out-strengths from this network to quantify both the degree to which an individual's directions of travel were sensitive to others (out-strength) and to which others were sensitive to them (in-strength), where both in- and out-strength are the sum of incoming or outgoing ties between individuals. We also calculated the centrality of each animal in the network, using alpha centrality. Alpha centrality is similar to eigenvector centrality but performs better in directed graphs where some nodes do not have incoming edges [22]. However, this measure of centrality was highly correlated with in-strength. Consequently, we report only the results for in-strength.

We adopted a core/periphery approach to measure network structure dependence [23]. Core/periphery structures are common features of flow-based networks, such as the spread of influence, and represent an intermediate-level network measure, in that the measures are not apparent solely at the local (one measure per node) or global level (one measure per network). The task of core/periphery algorithms is to classify nodes into successive cores and to identify the gradient between the core, comprising nodes that are highly connected to one another, and the periphery, which is made up of nodes that are connected to the core(s) but only loosely connected among themselves. Thus, if only a few interconnected individuals influence the movement of all members, this would result in a small single core and a large periphery. By the same token, no core would be detected if influence is diffuse and spreads across members in an unstructured way.

As our data are weighted and directed, we used a weighted extension of the k-core algorithm to classify nodes [24]. To estimate the magnitude of the core/periphery structure in the observed graph, we generated 10 000 Erdos Renyi graphs [25], using the same edge weights and number of nodes as the observed network. Nodes were assigned core values using the weighted k-shell algorithm. To compare the degree of core/periphery structure in the generated and observed graphs, we determined the degree of difference (*D*) in core values between all nodes, using the following equation:

2.3 $$D_{\mathrm{core}}=\overset{n}{\underset{i=1}{\sum }}\overset{n}{\underset{j=1}{\sum }}|x_{i}-x_{j}|.$$

This provides an indication of the probability that any observed core/periphery structure is due to a chance arrangement of influence patterns. The ‘igraph’ [25] and ‘qgraph’ [26] packages were used for social network analysis and visualization.

## 3. Results

### 3.1. Estimated individual influence: direction matching

**Figure 2.**
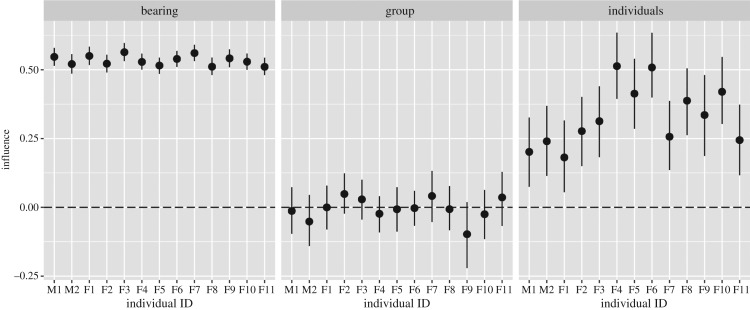
Estimated influence coefficients attributable to influences from the previous direction of travel (i.e. bearing), the mean group direction and the sum of the influence from directions to a specific individual in the group. Credible intervals around each point estimate are based on 99% highest posterior density intervals. Individuals on the *x*-axis are ordered according to rank, from highest ranked (M1) to lowest (F11).

When comparing the full model to the group-only model, we found that the full model had the lower WAIC value for each individual (table 1). Using the estimated coefficients from the full model for each individual, we identified the influence attributable to particular group members, the group as a whole and the previous direction of travel, highlighting the magnitude by which individuals were influenced by these specific factors (figure 2). On the whole, an animal's previous direction of travel had the largest influence (as one might expect, given that a large animal like a baboon has a certain amount of momentum, and any change in direction takes time to achieve), followed by the influence of particular group members and finally the influence of the group as a whole. In general, estimates of the 99% CI for the influence of the group as a whole contained 0. The WAIC model comparisons, and the uncertainty around the influence of the group as a whole, both suggest that individual locations play a larger role in predicting an animal's movement decisions than overall group orientation.

**Table 1.** Difference in WAIC values between the full model and the reduced group-only model for each adult animal. The difference (dWAIC) is calculated as the WAIC scores for the group-only model minus the full model. The uncertainty around the difference is presented in dSE.

individual	dWAIC	dSE
M2	107	22
F11	162	27
F2	260	34
F9	107	23
F4	412	43
F10	178	28
F8	209	32
F7	166	29
F6	340	39
F1	209	30
M1	189	28
F5	271	35
F3	294	36

### 3.2. Influence patterns at the individual level

**Figure 3.**
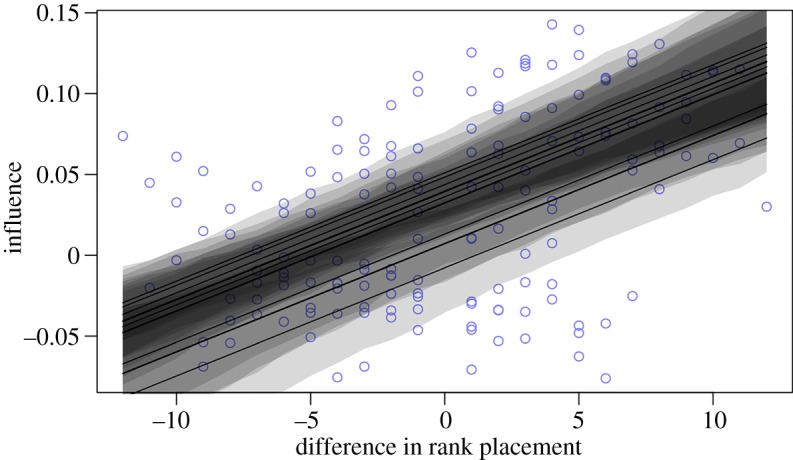
Model predicting the impacts of rank placement on influence between individuals. Each line represents the response of each individual, and the shaded region represents the uncertainty, and is calculated by the 95% highest posterior density interval.

Using rank difference to explain variation in influence estimates between individuals (equation (2.3)) indicates that a difference in one rank placement yielded a change of 0.007 in the estimated influence coeficient (99% CI = 0.003–0.010). As predicted, group members were therefore increasingly influenced by higher-ranking associates, and less so by lower-ranking associates (figure 3). Estimating *R*^2^ through posterior sampling [27] suggests that differences in rank explain 12% of the observed variation in influence between individuals, and that including varying intercepts (random effect of individual) increases the explained variance to 26%.

### 3.3. Influence patterns at the group level

**Figure 4.**
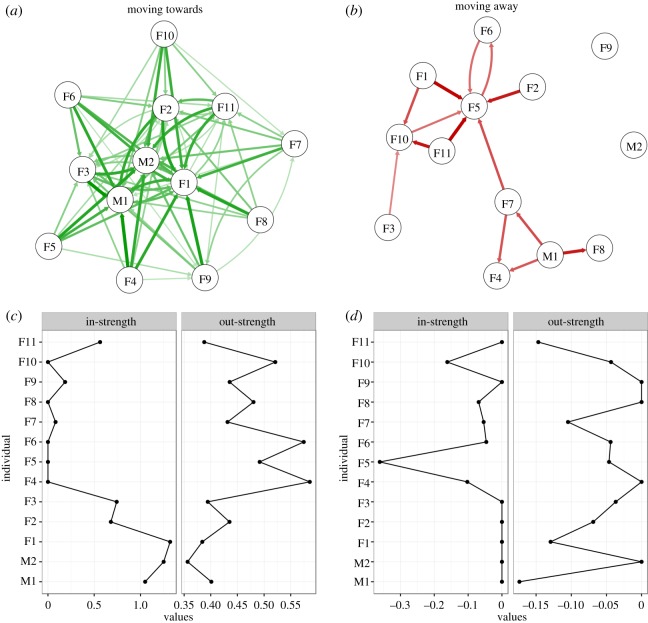
Individual-level movement bias visualized within a social network: (*a*) estimated positive influences when predicting the observed direction of travel (attractions) and (*b*) estimated negative influences when predicting the observed direction of travel (repulsions). The thickness of edges represents the edge weight, and arrows indicate the direction of the influence. In- and out-strength calculated from the attraction and repulsion networks are presented in (*c*) and (*d*), with individuals on the *y*-axis ordered according to the estimated rank.

Figure 2 presents the summed values of social influence, providing an overall estimate of individual influences. To gain a view of the interdependence between individuals, we then adopted a social network approach. When influence estimates at the individual level were used to develop a network, we found that animals showed marked variation in both in- and out-strength. When attractions were plotted against rank, in-strength was largely concentrated within higher-ranked individuals, whereas out-strength was highest at intermediate ranks (figure 4).

**Figure 5.**
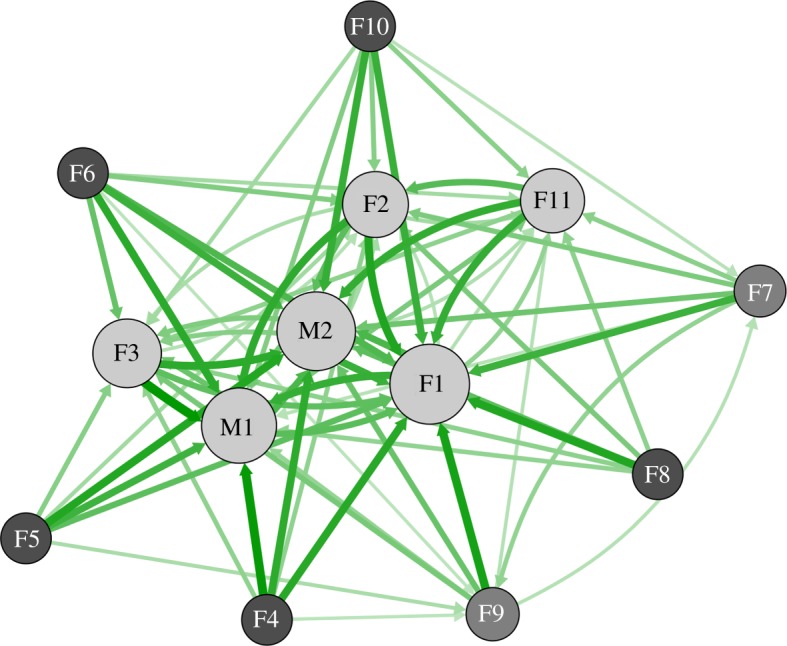
Core/periphery structure within the attraction network. Colours of individual nodes are based on the weighted k-shell algorithm (k-shell: dark grey = 0, medium grey = 1.00, light grey = 2.83), and edge colours and sizes are determined by attraction weights. Light grey nodes form the core, dark grey individuals form the periphery and large dark edges have high attraction weights. Node size represents the relative alpha centrality score within the group.

Against prediction, the group-level structure generated by individual-level attractions pointed to an inner core group with two shells (figure 5). The inner core was made up of the three highest-ranking females (F1, F2, F3), the males (M1 and M2) and a low-ranking female (F11). The shell closest to this core comprised two females (F7, F9), while the remaining individuals were categorized as peripheral. There was no core/periphery group-level structure associated with individual-level repulsions.

**Figure 6.**
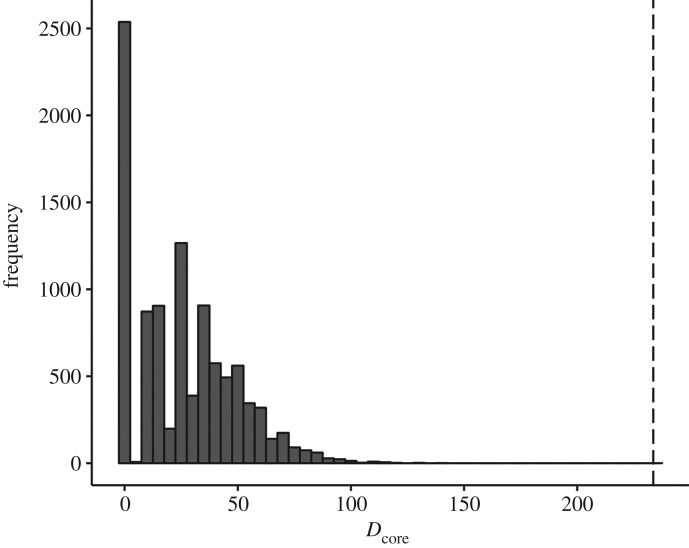
Histogram of 10 000 permutations of the observed graph. Permutations were based on randomly reassigning observed weighted edges to a graph with the same number of nodes. *D*_core_, a measure of the magnitude of core/periphery structure, was calculated using equation (2.3). The *D*_core_ value for the observed graph was 233.59 (dashed line).

When compared to permuted graphs, the magnitude of core/periphery structure in the observed attraction network exceeded the 95% quantile of the generated graphs (figure 6), with the *D*_core_ measure of the observed data (233.59) falling outside the 95% quantile of the permuted graphs (66.48). This suggests that the structuring in the network, in terms of core/periphery, was larger than would be expected from chance alone. That is, it is very unlikely that the core/periphery structure we see here could be generated at random (i.e. by rearranging attractions without reference to identity).

### 3.4. Influence patterns extracted from a simulated group

Given the interdependent nature and somewhat low resolution of our movement data, we used simulations to aid in interpreting our results (electronic supplementary material, A). We find two main results from these simulations which guide our subsequent interpretations. The first is that our simulations show that, given the resolution of our data, we cannot gain precise estimates of which animals actively follow each other. That is, if A follows B, for example, and B follows C, then our method will estimate that C has an influence on A, but will not necessarily pick up that this occurs exclusively via B. Thus, our results should be interpreted as indicating which animals show a systematic influence on others' movements (or conversely are influenced by the movements of others), but we cannot estimate the precise manner by which this occurs. We should also note, however, that this is not purely a matter of data resolution. In our simulations, we specified whom each animal followed, i.e. we knew the ‘mind’ of our agents. In the real world, however, we cannot know this *a priori*. Hence, even if a direct influence is found between A and C, if they are always found near B, one still cannot establish that A is sensitive to C specifically. It is possible to do so empirically by investigating instances when B and C move in different directions and detecting whether A systematically follows one and not the other; it is here that the advantages of high-resolution data are to be found—instances may occur more frequently in such datasets providing a sample of sufficient size to detect this effect. If, however, the situation never arises and A, B and C are always found together, then even high-resolution data will not allow a precise estimate of whether A is directly following B or C. It is also useful to note that, even in our simulations, when we designate that A is directly influenced by B and not C, it remains the case that C has an indirect influence on A. Thus, it would also be misleading to state that, even in our simulations, movement bias occurs solely at the dyadic level. In sum, then, our data should be interpreted as showing the relative degree of influence that animals exert on each other, but we cannot claim to show that a given animal has the goal of following a particular individual.

The second aspect of our simulations that aid in interpreting our results is that the estimate of the core, given the ‘downstream’ effects described above, tends to be over-inclusive, i.e. the core contains more animals than is actually the case. We estimate the ability of the core/periphery algorithm to correctly identify network structure using 100 h of simulated data (electronic supplementary material, S1). As our field data consist of approximately 700 h, the simulations represent a lower bound estimate of the abilities of the core/periphery algorithm. Our simulations also revealed, however, that the combination of core/periphery structure plus centrality measures can help distinguish the presence of a core/periphery structure, as opposed to the presence of a single influential individual (i.e. as in a despotic leadership model) (electronic supplementary material, S1), or a completely diffuse system (i.e. where individual influence is homogenous). Thus, our data are of sufficient resolution to investigate the question of interest.

## 4. Discussion

Our results show that, among our baboons, individuals displayed consistent patterns of movement bias towards or away from particular group members. When we compared directly the influence of individual animals versus the group as a whole, we found that animals' movements were more heavily influenced by specific animals than by the trajectory of the group. This supports the idea that, in social groups on the move, identity matters, and that individual identity is therefore necessary for extracting/testing the behavioural rules of collective movement.

Additionally, we found that individuals showed heterogeneous behavioural responses. As predicted, we found an influence of rank on movement patterns: larger rank differences between animals resulted in higher estimates of movement bias, with the lower-ranking individuals more influenced by higher-ranking individuals. In addition to this effect of rank, we also found clear individual differences: some individuals were much more influential than others, and their relative position in space exerted a large influence on the movement decisions of other animals (in-strength, figure 4*c*). Similarly, we found that some individuals were more heavily influenced by others (out-strength, figure 4*c*). These consistent differences at the individual level may point to differences in responsiveness, perhaps reflecting individual differences in temperament [28].

When we examined the combined interdependence of individuals, we found that social structures at the level of the group emerged. Contrary to our prediction, our results suggested a core/periphery structure, with a core set of largely high-ranking individuals exerting an influence on the movement decision-making of other lower-ranking animals. Given our simulated tests, we expect membership in the core to be a coarse estimate; nevertheless, the presence of a gradient in influence between a highly interdependent core and a disconnected periphery is apparent and unlikely to be due to chance. The heterogeneity of individual influences also points in this direction, and allows us to exclude the notion of a single leader or a completely diffuse influence structure. This discrepancy with the findings of Stueckle & Zinner's [8] analysis may reflect the fact that the current study considered animals while they were on the move, rather than initiating a departure from a sleeping cliff, and/or it may reflect differences in methods. Having said this, the core clearly contains more than one individual, and in that sense shows a more distributed pattern of influence than a despotic leadership model, which is the alternative that Stueckle & Zinner [8] tested. Overall then, these results suggest that heterogeneities in behavioural responses at the individual level lead to group-level social structures in our troop.

The presence of a core structure within a network of dependencies (peripheral nodes) has been shown to produce relevant group-level behaviours [23]. For example, a set of highly connected individuals in a core can make the group more resistant to the removal of any particular individual, enhancing the robustness of group-level behaviour [23] (e.g. the death of one individual in the core does not lead to breakdown of the existing group influence structure). At the same time, diversity in core group membership can lead to group-level behavioural flexibility [23]. For example, core members with differential knowledge of resource availability allow the group to benefit from multiple sources of information, and may increase group foraging efficiency under variable or changing environments, as those with the most influence on group movement have diverse knowledge to draw on. The size of the core is also relevant: the larger the number of animals in the core, the more likely it is that decision-making within the core will be shared, reducing the ability of any one individual to assert its will without compromise on the group as a whole [23].

Comparison of multiple social groups across different environments could identify possible consequences or adaptive advantages of this kind of influence structure in relation to environmental and social conditions. For example, using the concept of core/periphery structures, do groups with larger cores show more flexible/adaptable behaviour? Are there consequences to having a core that is too large (i.e. too much behavioural diversity in core individuals)? Are there conditions under which we might expect multiple cores, or decreased gradients between periphery and core? We might also expect more heterogeneous groups (e.g. body size, age, sex) to present conditions under which shared decision-making might be advantageous [29]: are larger core structures associated with more heterogeneous groups, and if so under what conditions? This approach focuses less on identifying a ‘leader’ [30], and more on quantifying the structure of influence within mobile social groups.

Our results, however, cannot speak to how this structure is formed or maintained [31]. One possibility is that animals learn that the movement of a particular individual is strongly correlated with the movement of the entire group (i.e. individual learning), and tracking another individual is a simple way to maintain contact with the group as a whole. Another possibility is that individuals are followed consequent on past social interactions (e.g. following a particular individual simply leads to favourable conditions), or that animals copy others' behaviour (i.e. mimetism). It is also possible that the core group is followed due to the particular characteristics of the individuals comprising the core, above and beyond the influences of rank tested here. In the case of both individual and social learning, the formation of the observed influence structure might be a result of individual-level adaptation to changes in group-level characteristics. This can be viewed as a form of dynamical system in which individual- and group-level patterns result from individual behaviour leading to group-level patterns, and group-level patterns then influence individual behaviours. This type of dynamical system, composed of adaptable individuals both creating and being formed by their interdependence, represents a challenge to current methodology, yet lies at the heart of many interesting problems. We suggest that mobile social troops offer an excellent model system in which to better understand these dynamical patterns; their development, maintenance and responsiveness to environmental conditions [1,32].

## Supplementary Material

Simulated results

## Data accessibility

Baboon movement data are available on Movebank [33] and Dryad [34]; r code used in the analysis is available from github (github.com/tbonne/Rcode_Baboon_Movement_Bias/).
