# Supplementary material for: Individual-level movement bias leads to the formation of higher-order social structure in a mobile group of baboons
Source: R Soc Open Sci. 2017 Jul 12;4(7):170148. doi: 10.1098/rsos.170148 (PMC5541535; doi:10.1098/rsos.170148)
Supplement: Simulated results [file rsos170148supp1.docx]

**Supplementary A: Simulation tests**

We simulate movement data with a known social influence structure to assess whether our data can provide information about the underlying pattern given our temporal resolution (mean revisit time = 9 min) and errors introduced using interpolation. The simulated group followed Bonnell et al. [1] where individuals forage, moving to food patches, and when “full” move towards their chosen social partner. This provides a framework whereby the individual in question is only showing the behaviour of interest at certain times (moving towards social partner) and otherwise is moving based on availability of local resources.

We simulate a moving troop of 12 individuals with alternative social influence structures, and extract 1 sec resolution over a period of 100 h. As our observed data is ~700 h, we view this simulation test as the lower end of what is achievable with the direction matching method. We degrade the simulated data to a temporal resolution by resampling from lognormal distribution of revisit times fit to the revisit times in the observed data [1]. We then use interpolation to estimate group member positions at observations of any given focal animal. Each animal in turn is treated as the focal animal and Eq. 1 is used to extract estimates of movement bias. These influences are then visualized using a social network, and core periphery measures are estimated.

Below we examine three different simulations. The first simulates a troop which has a sub-set of individuals who are interdependent and are followed by others outside this subset (i.e., a troop with a core-periphery structure). The second simulates a troop with only one leader, all others in the group follow this leader (i.e., despotic movement model). The last simulation examines the case where the temporal resolution of the data is still low (average revisit time of 10min) but where interpolation error is removed. The first two simulations test whether we can distinguish between core-periphery structure and despotic groups, whereas the last simulation investigates our proposed methods under conditions of low spatial error (e.g., group snapshots). We further test two methods in fitting Eq. 1 to the data: linear regression, and circular regression.

**1. Linear method**

To estimate movement bias from the simulated datasets we first attempt a linear method in which the x and y components of each observation are treated as independent. This assumption allows for the creation of a matrix in which each observation contributes two rows of data. A linear regression approach is then used to estimate the beta values that reduce error between observed movement in both x and y dimensions.

*1.1. Simulated social influence structure with a core:*

We simulate group movement of a troop with three individuals in a core and 6 individuals on the periphery (Fig. S1).


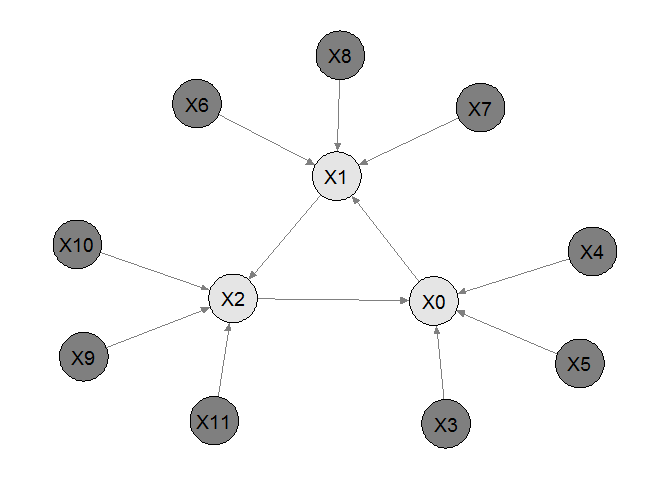


Figure S1: Actual influence structure in the simulated group. Direction of arrows signify who is following whom. Node colour corresponds to the core-periphery grouping, with light grey colour for the core and increasingly darker colour towards the periphery.

We find that the most central individuals are those that are expected given the actual influence structure. When we examine overall in-strength, we find that individuals 0, 1, and 2, are correctly identified as having high in-strength relative to the other group members (Fig. S2). Out-strength does show some variability, but without a clear trend (Fig. S2). We find that the group mean direction is correctly identified as a non-influential factor, and that individuals are not avoiding each other (i.e., no repulsion estimated in the simulated data). However, we find that, with the reduced temporal resolution and error introduced by the interpolation, we do not fully reproduce the simulated influence structure (Fig. S3). We find that the positions of individuals “downstream” of particular dyadic attractions provide information for the model predicting direction of travel. For example, individual 6 is influenced only by individual 1 in the simulations, yet in the reduced dataset we find that the model estimates that individual 6’s direction of travel can also be explained by the locations of 0, 1, 2, 7, and 8. As individuals 0, 1, and 2 are all mutually dependent, it is perhaps not surprising that 6 is found to be influenced by 0, 1, and 2. Similarly, 7 and 8 are both influenced by 1 so it is not surprising that their location provides some predictability given that 6 is also influenced by 1. This suggests that we cannot expect our data to estimate specific dyadic interactions independently of their interrelations with other group members. Hence, we can only estimate a group-level view of the influence structure. Similarly, when we examine the ability of the core-periphery algorithm to correctly identify the core, we find that the estimates includes all true core nodes, but also include some that are not in the simulated core. This suggests again that with our lower resolution dataset, we are likely only to gain a group level estimate of the influence structure (Fig. S3).


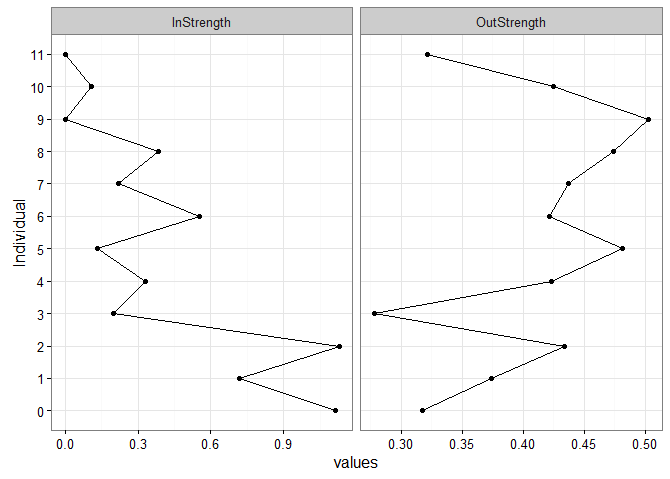


Figure S2: Estimated in- and out-strength from the simulated data.


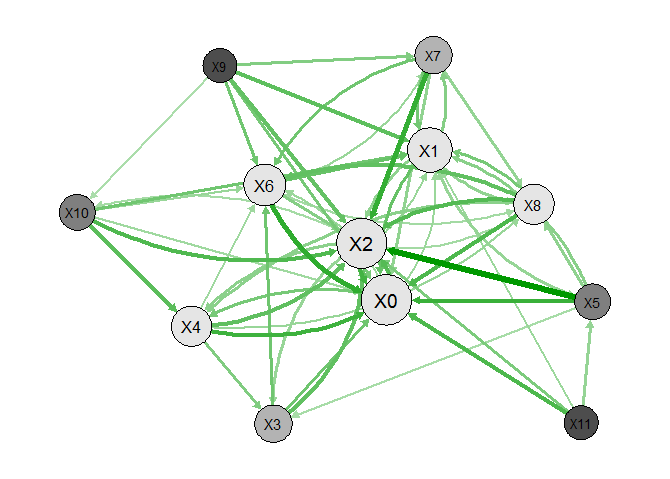


Figure S3: Estimated influence structure of the simulated group. The size and greenness of arrows represent the strength of influence, and the direction of the arrows signify who is influenced by whom. The size of the nodes correspond to the relative alpha centrality of the node. The colour of the node corresponds to the core-periphery grouping, with light grey colour for the core and increasingly darker grey colour towards the periphery.

Permutation of edges suggest that the amount of core periphery structure is larger than that expected by chance arrangement (Fig S4).


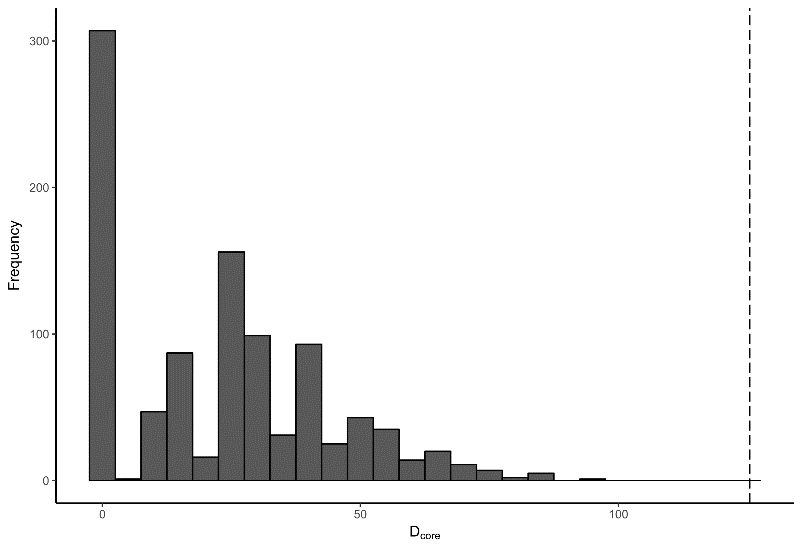


Figure S4: Histogram of the amount of core periphery structure generated from 1000 permutations; upper 95% confidence interval 60.63. The structure estimated from the simulated movement data was 125.45 (dashed vertical line).

*1.2. Simulated influence structure with one central individual:*

We simulate group movement of a troop with one individual acting as a leader, all others in the group follow this individual, whereas the leader follows no one (Fig. S5).


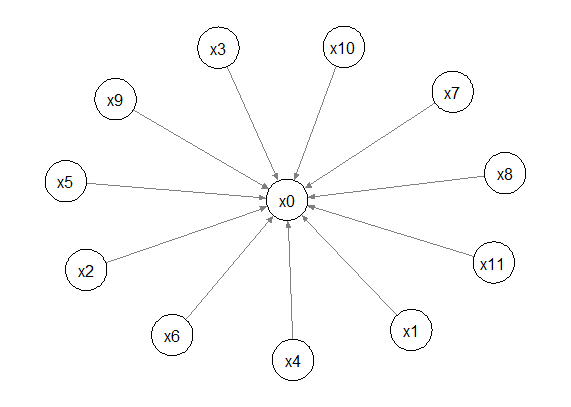


Figure S5: Actual influence structure in the simulated group. Direction of arrows signify who is following whom. No core-periphery structure is found, as there is only one individual leading all others.

We find that, again, the estimates correctly identify the central individual. When looking at in- and out-strength, the estimates correctly identify the leader as the individual with high in-strength from others and low out-strength to others (Fig. S6) thus correctly predicting no influence bias from any individual in the group (i.e., out-strength = 0). However, we again find that with our temporal resolution and estimated errors from interpolation, we are not likely to reproduce the actual influence structure between individuals. As with the previous simulation, we find that the core-periphery algorithm overestimates the size of the core. However, in combination with the in- and out- strength measures it is possible to distinguish between a troop lead by a single leader and one led by core of interdependent individuals. This suggests that a combination of centrality measures and core-periphery analyses can give a more accurate picture than core-periphery estimates alone (Fig. S7).


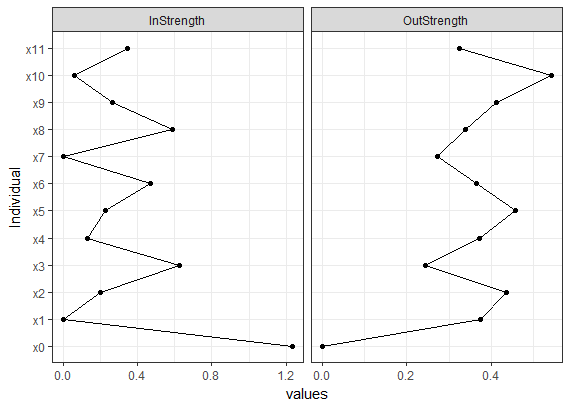


Figure S6: Estimated in- and out-strength from the simulated data.


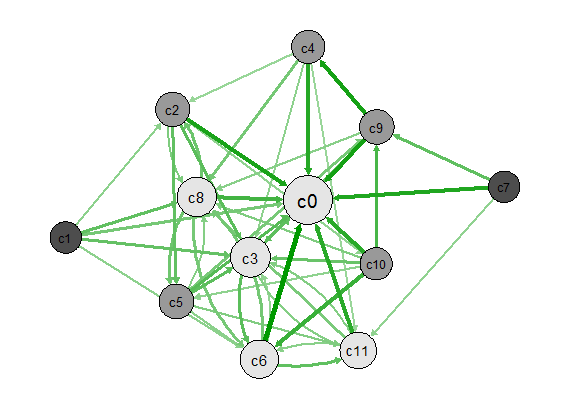


Figure S7: Estimated influence structure of the simulated group. The size and greenness of arrows represent the strength of influence, and the direction of the arrows signify who is influenced by whom. The size of the nodes correspond to the relative alpha centrality of the node. The colour of the node corresponds to the core-periphery grouping, with light grey colour for the core and increasingly darker grey colour towards the periphery.

*1.3. Simulated influence structure with one central individual (no interpolation error):*

We additionally test the direction matching approach with the despotic movement model in absence of interpolation error. This mimics the case where an observer can take very accurate snapshots of individual locations sequentially throughout an observation period: an observation method that is facilitated by handheld or animal collared GPS. We find that with the relatively low temporal resolution of our data (~10min revisit times), in the absence of interpolation noise, the direction matching method correctly estimates the simulated social influence structure (Fig. S8). Using these estimates the core-periphery algorithm similarly finds no core-periphery structure (Fig. S9). This suggests that future studies interested in extracting social influence structures from movement data should focus on reducing spatial uncertainty, and that relatively low temporal resolution can be sufficient.

**
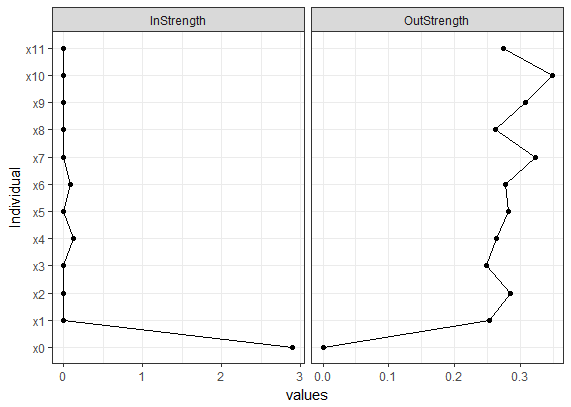
**

Figure S8: Estimated in- and out-strength for the simulated data.


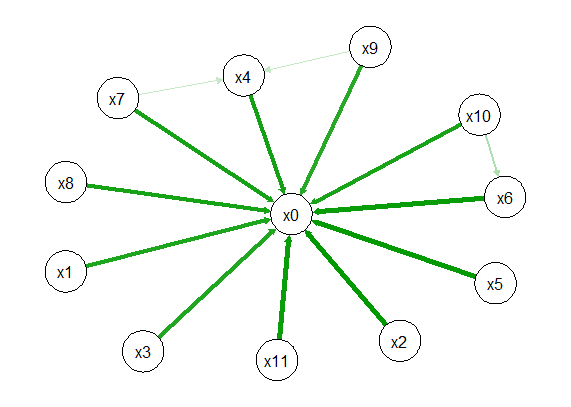


Figure S9: Estimated influence structure of the simulated group. The size and greenness of arrows represent the strength of influence, and the direction of the arrows signify who is influenced by whom. No core-periphery structure is found in the simulated group.

**2. Circular method**

To estimate movement bias from the simulated datasets, we used a circular regression following Rivest et al. [2]. This method uses the arctan2 as a link function for the von Mises distribution, allowing both x and y components of each observation to be treated simultaneously (i.e., each observation produces only one row of data).

*2.1. Simulated social influence structure with a core:*

We simulate group movement of a troop with three individuals in a core and 6 individuals on the periphery (Fig. S10).


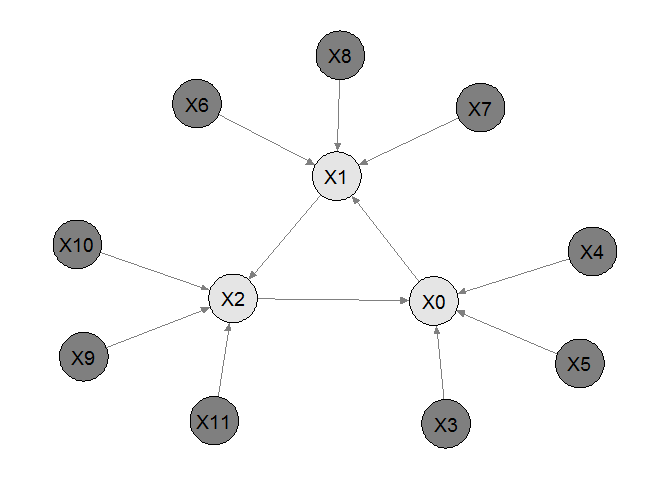


Figure S10: Actual influence structure in the simulated group. Direction of arrows signify who is following whom. Node colour corresponds to the core-periphery grouping, with light grey colour for the core and increasingly darker colour towards the periphery.

We find that the most central individuals are those that are expected given the actual influence structure. When we examine overall in-strength, we find that individuals 0, 1, and 2, are correctly identified as having high in-strength relative to the other group members (Fig. S11). Out-strength does show some variability, but without a clear trend (Fig. S11). We find that the group mean direction is correctly identified as a non-influential factor, except for two individuals, and that individuals are not avoiding each other (i.e., no repulsion estimated in the simulated data). However, we find that, with the reduced temporal resolution and error introduced by the interpolation, we do not fully reproduce the simulated influence structure (Fig. S12). We find that the positions of individuals “downstream” of particular dyadic attractions provide information for the model predicting direction of travel. For example, individual 6 is influenced only by individual 1 in the simulations, yet in the reduced dataset we find that the model estimates that individual 6’s direction of travel can also be explained by the locations of 0, 1, 7, and 8. As individuals 0 and 1 are mutually dependent, it is perhaps not surprising that 6 is found to be influenced by 0 and 1. Similarly, 7 and 8 are both influenced by 1 so it is not surprising that their location provides some predictability given that 6 is also influenced by 1. This suggests that we cannot expect our data to estimate specific dyadic interactions independently of their interrelations with other group members. Hence, we can only estimate a group-level view of the influence structure. Similarly, when we examine the ability of the core-periphery algorithm to correctly identify the core, we find that the estimates includes all true core nodes, but also include some that are not in the simulated core. This suggests again that with our lower resolution dataset, we are likely only to gain a group level estimate of the influence structure (Fig. S12).


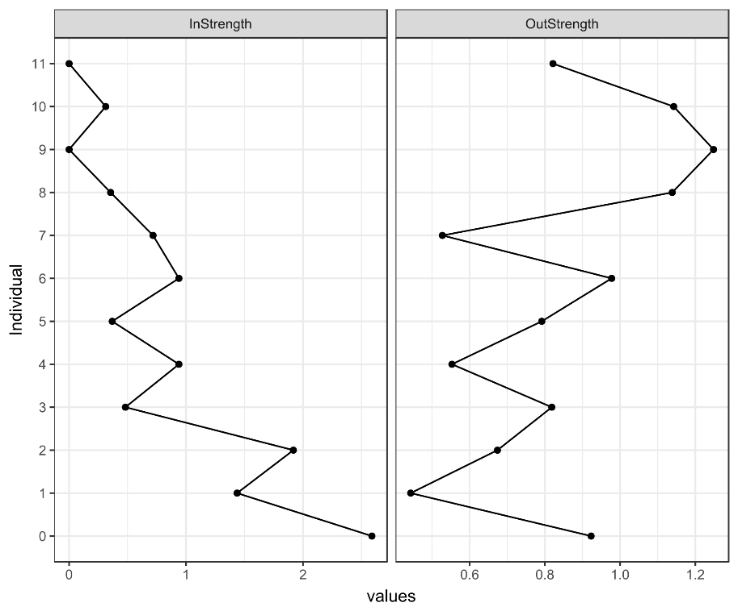


Figure S11: Estimated in- and out-strength from the simulated data.


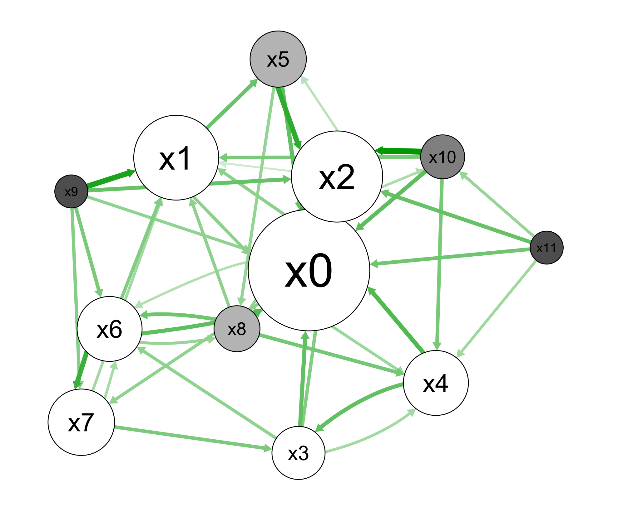


Figure S12: Estimated influence structure of the simulated group. The size and greenness of arrows represent the strength of influence, and the direction of the arrows signify who is influenced by whom. The size of the nodes correspond to the relative alpha centrality of the node. The colour of the node corresponds to the core-periphery grouping, with light grey colour for the core and increasingly darker grey colour towards the periphery.

Permutation of edges suggested that the amount of core periphery structure is larger than that expected by chance arrangement (Fig S13).


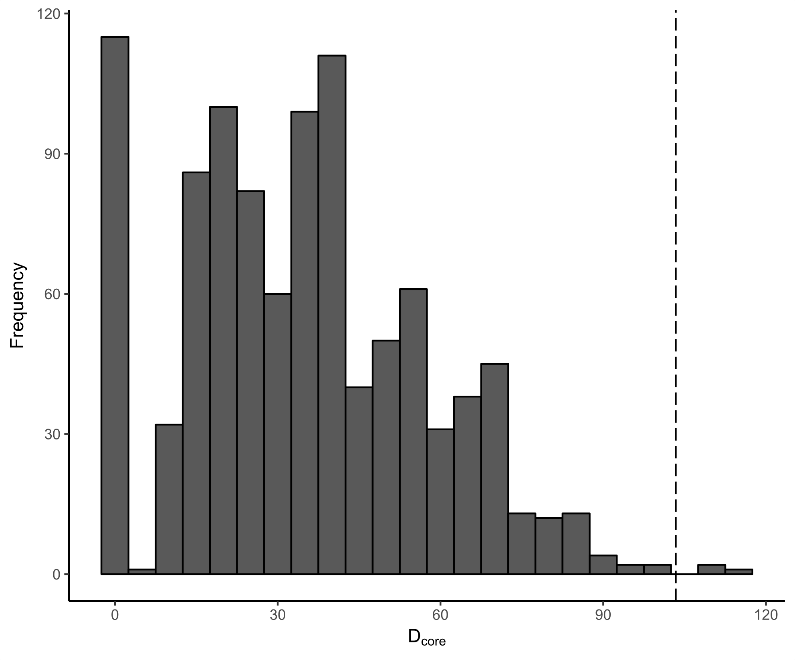


Figure S13: Histogram of the amount of core periphery structure generated from 1000 permutations; upper 95% confidence interval 72.36. The structure estimated from the simulated movement data was 103.37 (dashed vertical line).

*2.2. Simulated influence structure with one central individual:*

We simulate group movement of a troop with one individual acting as a leader, all others in the group follow this individual, whereas the leader follows no one (Fig. S14).


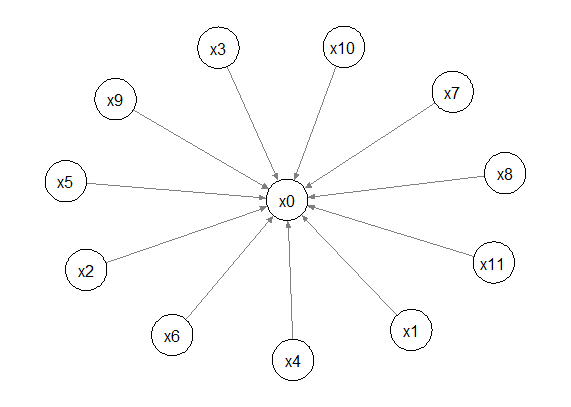


Figure S14: Actual influence structure in the simulated group. Direction of arrows signify who is following whom. No core-periphery structure is found, as there is only one individual leading all others.

We find that, again, the estimates correctly identify the central individual. When looking at in- and out-strength, the estimates correctly identify the leader as the individual with high in-strength from others and low out-strength to others (Fig. S15). However, we again find that with our temporal resolution and estimated errors from interpolation, we are not likely to reproduce the actual influence structure between individuals. As with the previous simulation, we find that the core-periphery algorithm over-estimates the size of the core (Fig. S16). However, in combination with the in- and out- strength measures it is possible to distinguish between a troop lead by a single leader and one led by core of interdependent individuals. This suggests that a combination of centrality measures and core-periphery analyses can give a more accurate picture than core-periphery estimates alone.


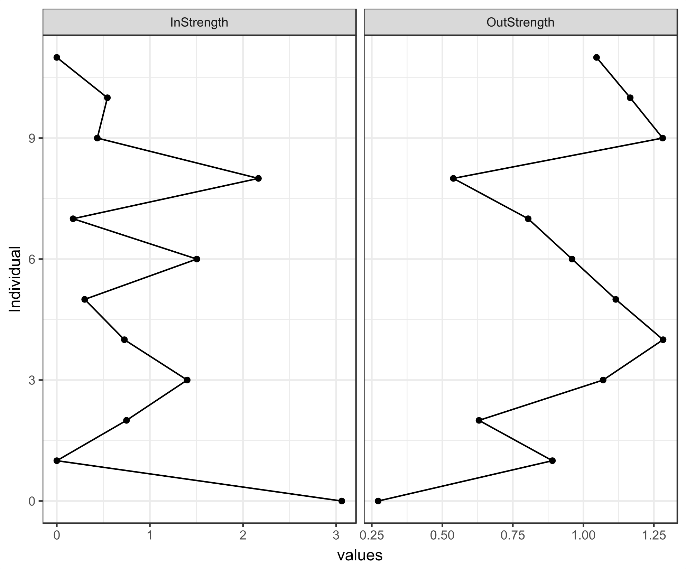


Figure S15: Estimated in- and out-strength from the simulated data.


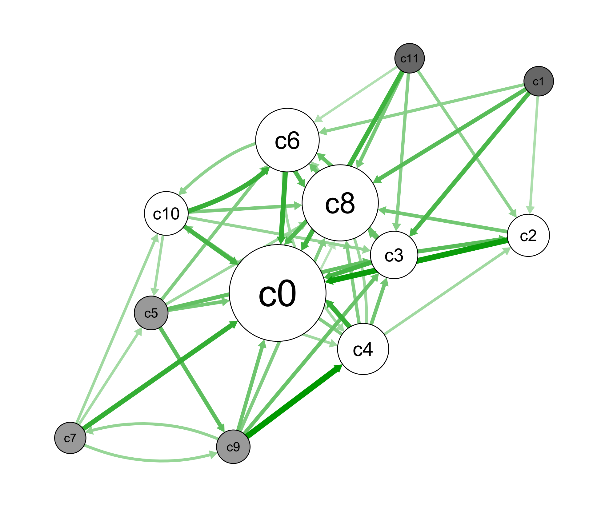


Figure S16: Estimated influence structure of the simulated group. The size and greenness of arrows represent the strength of influence, and the direction of the arrows signify who is influenced by whom. The size of the nodes correspond to the relative alpha centrality of the node. The colour of the node corresponds to the core-periphery grouping, with light grey colour for the core and increasingly darker grey colour towards the periphery.

Permutation of edges suggested that the amount of core periphery structure is only just larger than that expected by chance arrangement (Fig S17).


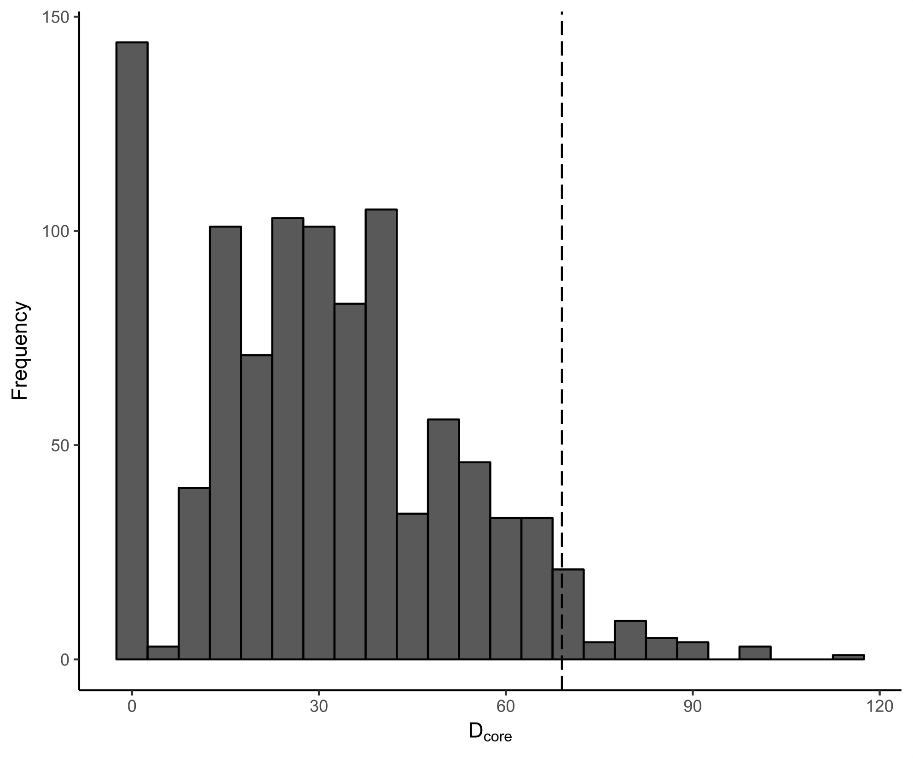


Figure S17: Histogram of the amount of core periphery structure generated from 1000 permutations; upper 95% confidence interval 66.26. The structure estimated from the simulated movement data was 68.99 (dashed vertical line).

*2.3. Simulated influence structure with one central individual (no interpolation error):*

We additionally test the direction matching approach with the despotic movement model in absence of interpolation error. This mimics the case where an observer can take very accurate snapshots of individual locations sequentially throughout an observation period, an observation method that is facilitated by handheld or animal collared GPS. We find that with the relatively low temporal resolution of our data (~10min revisit times), in the absence of interpolation noise, the direction matching method correctly estimates the simulated social influence structure (Fig. S18). Using these estimates the core-periphery algorithm similarly finds no core-periphery structure (Fig. S19). This suggests that future studies interested in extracting social influence structures from movement data should focus on reducing spatial uncertainty, and that relatively low temporal resolution can be sufficient.

**
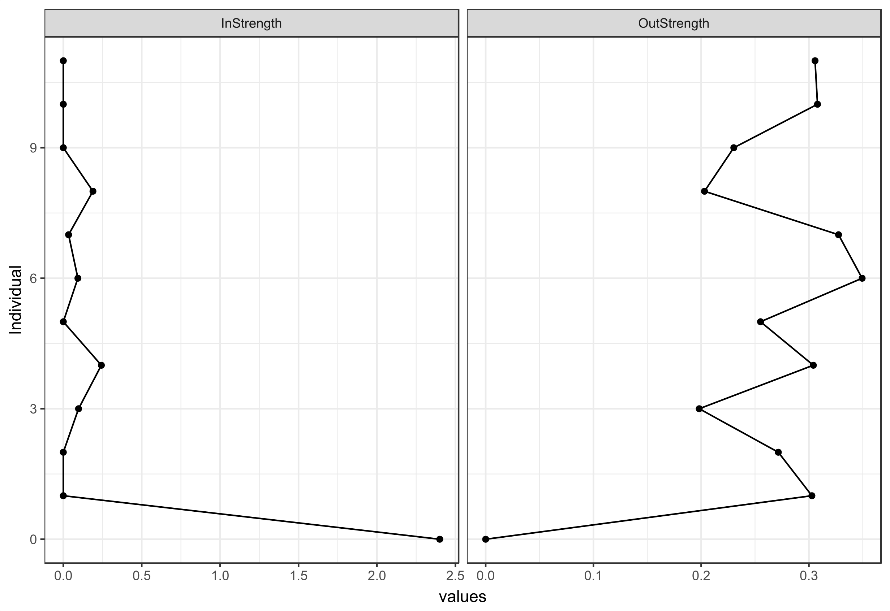
**

Figure S18: Estimated in- and out-strength for the simulated data.


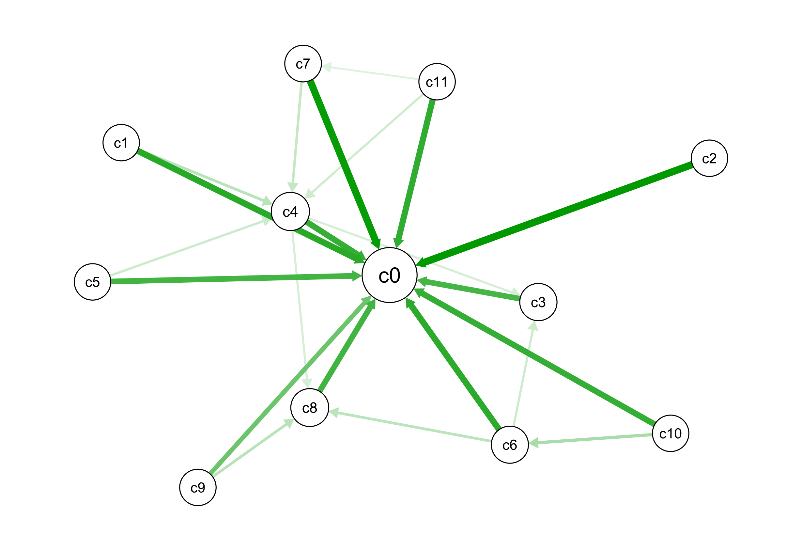


Figure S19: Estimated influence structure of the simulated group. The size and greenness of arrows represent the strength of influence, and the direction of the arrows signify who is influenced by whom. No core-periphery structure is found in the simulated group.

**Supplementary B: Main results using the circular method**

Below is a reproduction of the main results from the baboon dataset using the circular regression approach, as opposed to a linear regression.

*Estimated individual influence: direction matching*

When comparing the full model to the group-only model, we found that the full model had the lower WAIC value for each individual (Table S1), though standard errors around this difference suggest the group-only model is comparable for one individual (F9). Using the estimated coefficients from the full model for each individual, we identified the influence attributable to particular group members and the group as a whole, highlighting the magnitude by which individuals were influenced by these specific factors (Fig. S20). On the whole, specific group members had the largest influence, followed by the influence of the group as a whole. For all but two individuals, estimates of the 99% CI for the influence of the group as a whole contained 0. The WAIC model comparisons and the uncertainty around the influence of the group as a whole both suggest that individual locations play a larger role in predicting an animal’s movement decisions than overall group orientation.

Table S1: Difference in WAIC value between the full model and the reduced group-only model for each adult animal. The difference (dWAIC) is calculated as the WAIC scores for the group-only model minus the full model. The uncertainty around the difference is presented in dSE.

| **Individual** | **dWAIC** | **dSE** |
| --- | --- | --- |
| M2 | 18.5 | 7.0 |
| F11 | 34.5 | 10.4 |
| F2 | 37.0 | 10.2 |
| F9 | 9.0 | 8.0 |
| F4 | 111.5 | 17.4 |
| F10 | 54.1 | 13.7 |
| F8 | 71.2 | 14.0 |
| F7 | 69.0 | 13.0 |
| F6 | 124.4 | 30.6 |
| F1 | 39.1 | 9.3 |
| M1 | 23.0 | 8.4 |
| F5 | 87.4 | 19.5 |
| F3 | 70.5 | 12.1 |


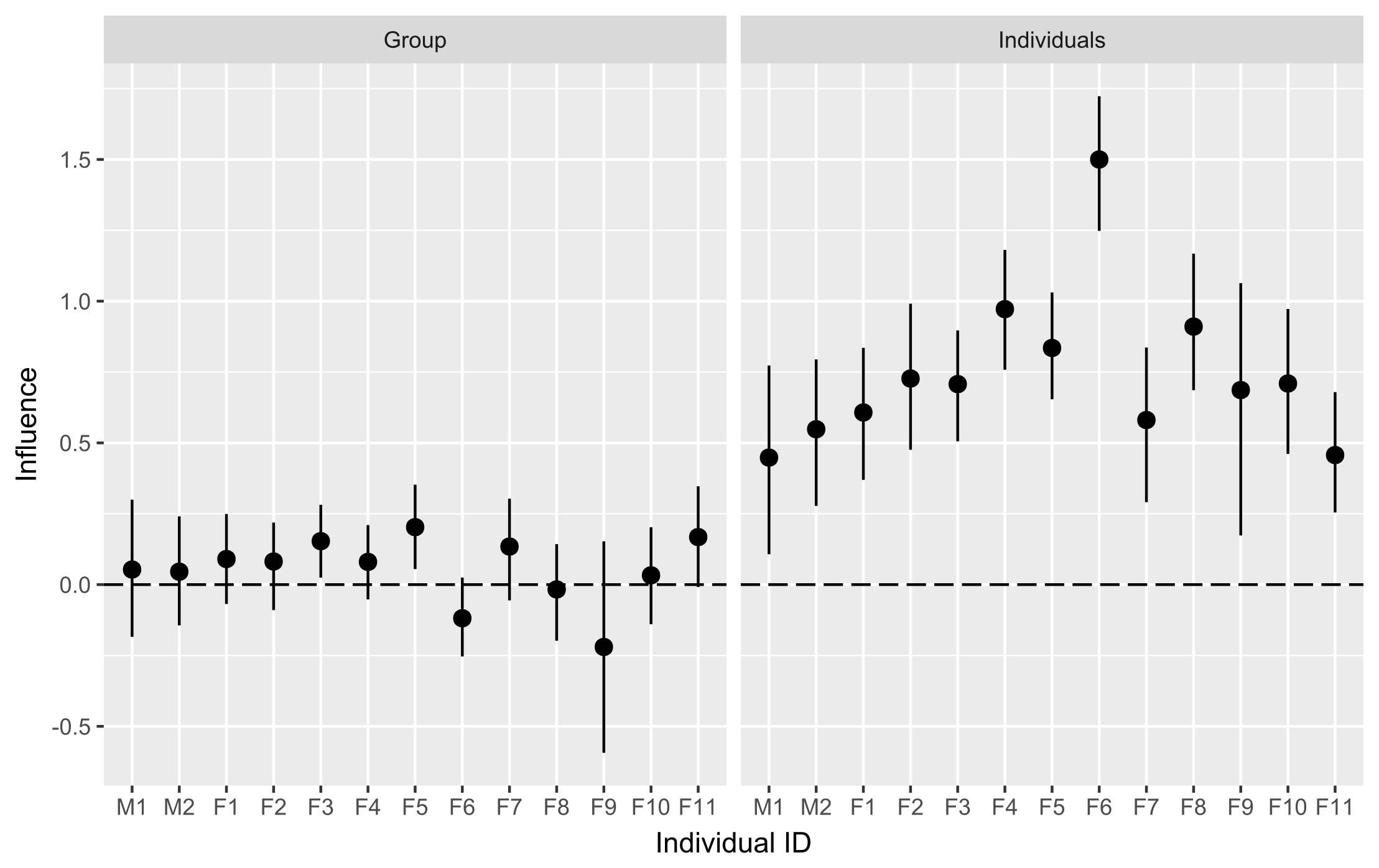


Figure S20: Estimated influence coefficients attributable to influences from the mean group direction, and the sum of the influence from directions to specific individual in the group. Credible intervals around each point estimate are based on 99% HPDI. Individuals on the x-axis are ordered according to rank, from highest ranked (M1) to lowest (F11).

*Influence patterns at the individual level*

Using rank difference to explain variation in influence estimates between individuals (eq.3) indicates that a difference in one rank placement yielded a change of 0.004 in the estimated influence coeficient (99% CI = 0.001 - 0.08). As predicted, group members were therefore increasingly influenced by higher-ranking associates, and less so by lower-ranking associates (Fig. S21). Estimating R^2^ through posterior sampling [3] suggests that differences in rank only explains 4% of the observed variation in influence between individuals, and that including varying-intercepts (random effect of individual) increases the explained variance to 7%.


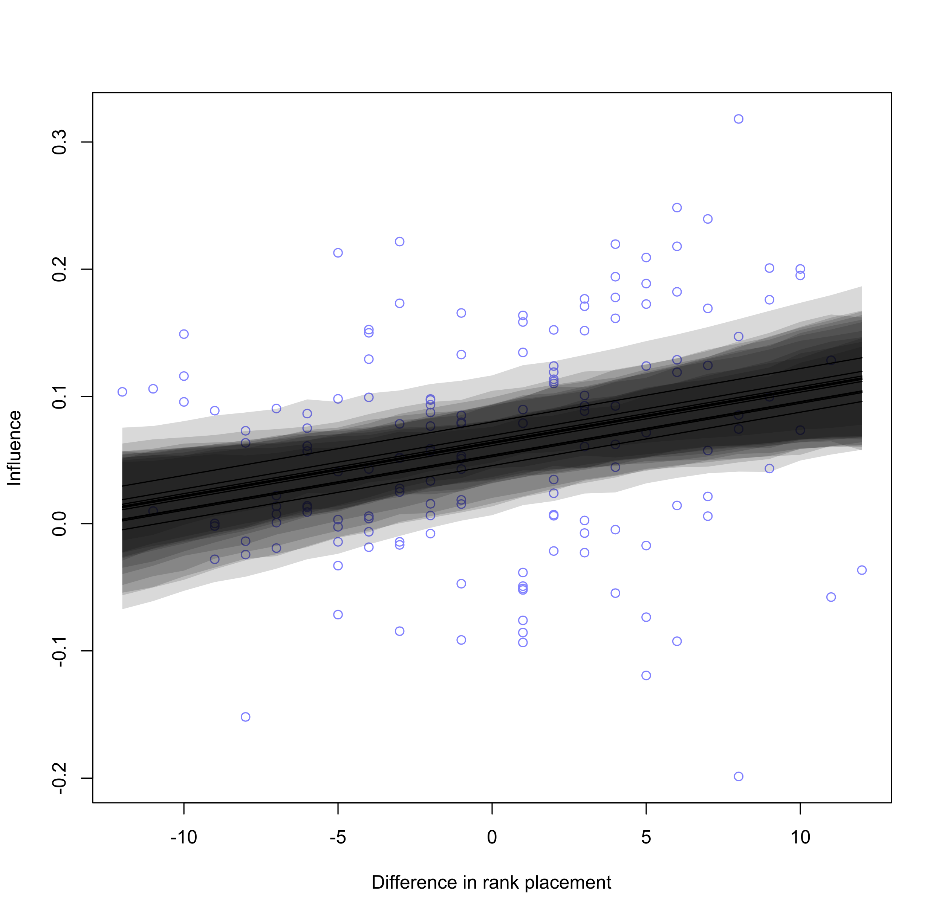


Figure S21: Model predicting the impacts of rank placement on influence between individuals. Each line represents the response of each individual, and the shaded region represents the uncertainty, and is calculated by the 95% HPDI.

*Influence patterns at the group level*

Figure S20 presents the summed values of social influence, providing an overall estimate of individual influences. To gain a view of the inter-dependence between individuals, we then adopted a social network approach. When influence estimates at the individual level were used to develop a network, we found that animals showed marked variation in both in- and out-strength. When attractions were plotted against rank, in-strength was largely concentrated within higher ranked individuals, whereas out-strength was highest at intermediate ranks (Fig. S22).


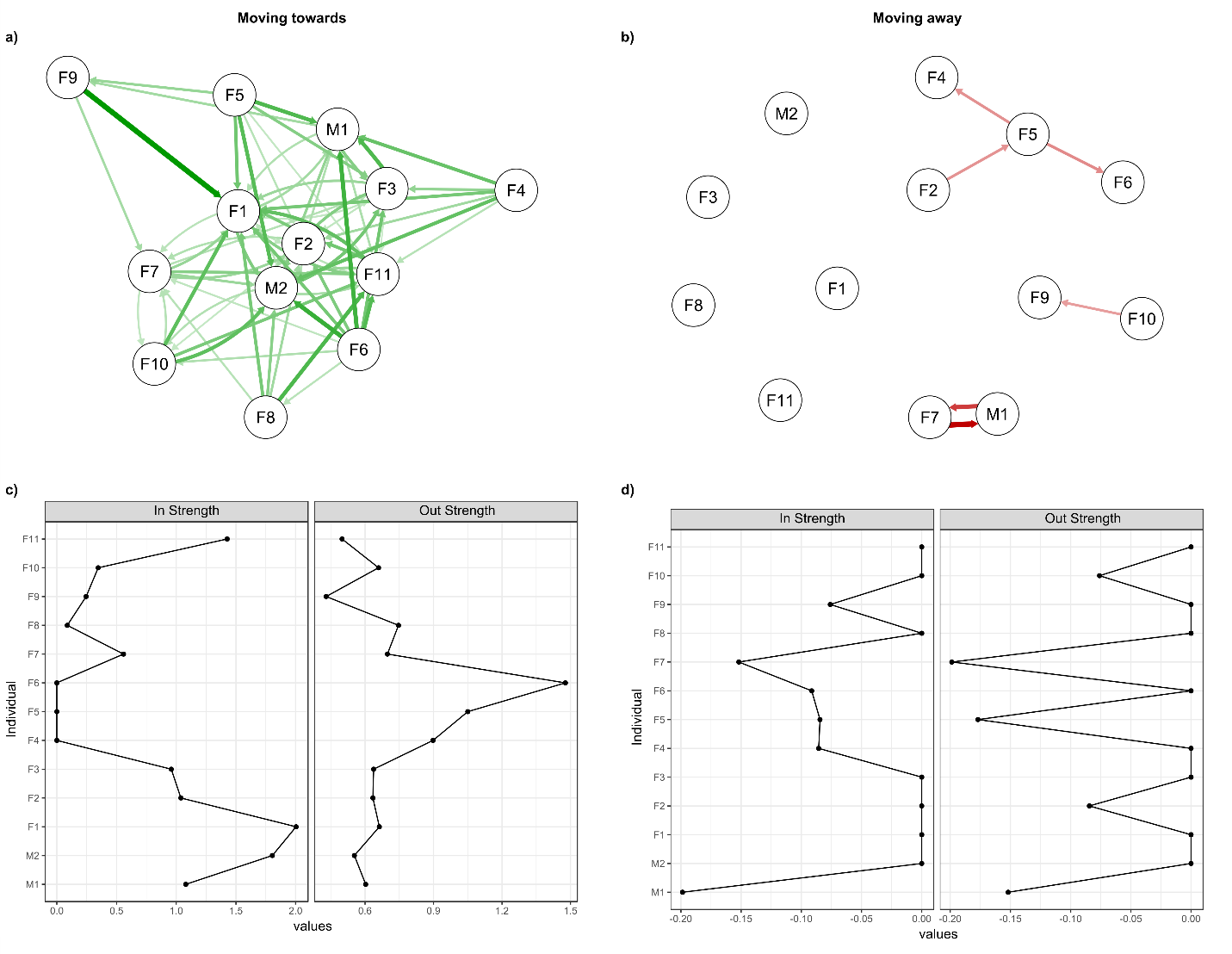


Figure S22: Individual level movement bias visualized within a social network: a) estimated positive influences when predicting observed direction of travel (attractions), and b) estimated negative influences when predicting observed direction of travel (repulsions). The thickness of edges represents the edge weight, and arrows indicate direction of the influence. In and out strength calculated form the attraction and repulsion networks are presented in c) and d), with individuals on the y-axis ordered according to estimated rank.

Against prediction, the group-level structure generated by individual-level attractions pointed to an inner core group with two shells (Fig. S23). The inner core was made up of the three highest-ranking females (F1, F2, F3), the males (M1 and M2), and a low ranking female (F11). The shell closest to this core comprised three females (F7 and F10), flowed by a shell composed of one female (F9), while the remaining individuals were categorised as peripheral (F4, F5, F6, F8). There was no core-periphery group-level structure associated with individual-level repulsions.


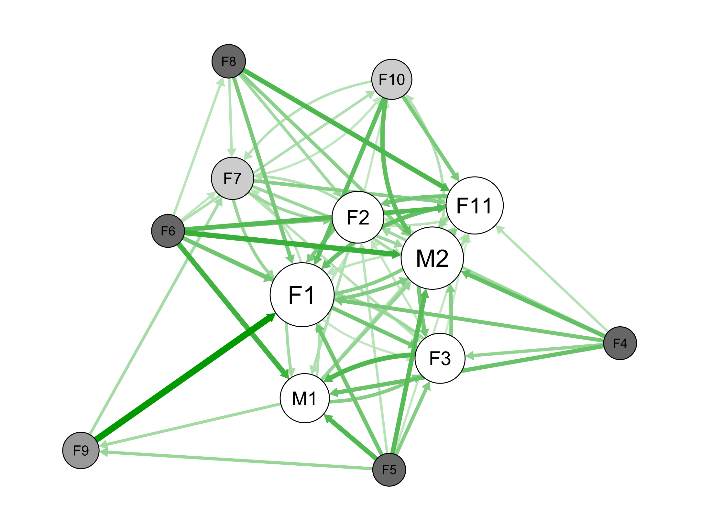


Figure S23: Core-periphery structure within the attraction network. Colours of individual nodes are based on the weighted k-shell algorithm (k-shell: dark grey=0, medium grey=1, light grey=1.73, white=2.45) and edge colours and sizes are determined by attraction weights. White nodes form the core, dark grey individuals form the periphery, and large dark edges have high attraction weights. Node size represents the relative alpha centrality score within the group.

When compared to permuted graphs, the magnitude of core/periphery structure in the observed attraction network exceeded the 95% quantile of the generated graphs (Fig. S24), with the D_core_ measure of the observed data (190.83) falling outside the 95% quantile of the permuted graphs (72.06). This suggests that the structuring in the network, in terms of core/periphery, was larger than would be expected from chance alone. That is, it is very unlikely that the core-periphery structure we see here could be generated at random (i.e., by rearranging attractions without reference to identity).


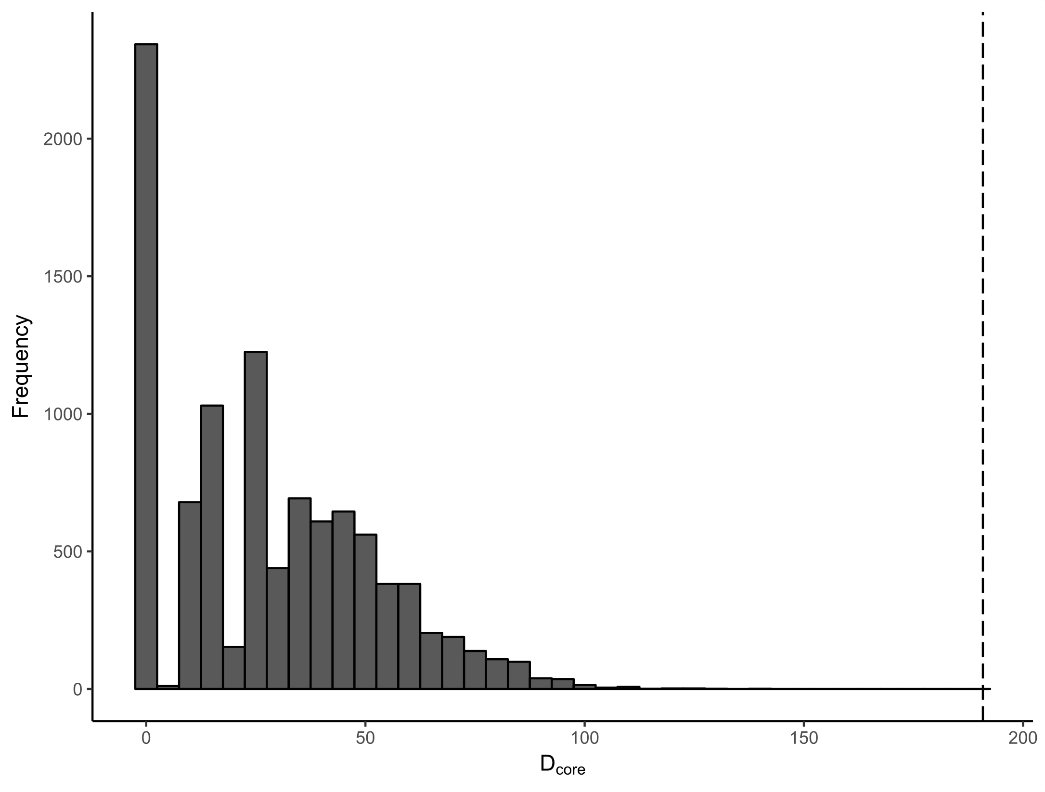


Figure S24: Histogram of 10,000 permutations of the observed graph. Permutations were based on randomly re-assigning observed weighted edges to a graph with the same number of nodes. D_core_, a measure of the magnitude of core-periphery structure, was calculated using eq. 3. The D_core_ value for the observed graph was 190.83 (dashed line).

**Supplementary C: Results combining individual direction and distance measures**

To examine the effects of both direction and distance of particular individuals on a focal animal’s movement we present a model comparisons analysis. We run three models: 1) using equation 1 in our manuscript where there is no influence of distance, 2) using equation 1 again but weighting the effect of the group by the magnitude of directional agreement of group members (${cv}_{t}$), measured as the circular variation in angles to group members, and finally 3) where the influence of particular individuals on the focal animal’s movements is weighted by distance ($d_{i,j}$).

| ${\hat{\boldsymbol{v}}}_{t}=\sum_{i\neq j}^{n} \beta_{i,j}{\hat{\boldsymbol{v}}}_{i,j}+\beta_{cm}{\hat{\boldsymbol{v}}}_{cm}+\beta_{t-1}{\hat{\boldsymbol{v}}}_{t-1}$ | Eq.1 |
| --- | --- |
| ${\hat{\boldsymbol{v}}}_{t}=\sum_{i\neq j}^{n} \beta_{i,j}{\hat{\boldsymbol{v}}}_{i,j}+\frac{\beta_{cm}{\hat{\boldsymbol{v}}}_{cm}}{{cv}_{t}}+\beta_{t-1}{\hat{\boldsymbol{v}}}_{t-1}$ | Eq. 2 |
| ${\hat{\boldsymbol{v}}}_{t}=\sum_{i\neq j}^{n} \frac{\beta_{i,j}{\hat{\boldsymbol{v}}}_{i,j}}{d_{i,j}}+\frac{\beta_{cm}{\hat{\boldsymbol{v}}}_{cm}}{{cv}_{t}}+\beta_{t-1}{\hat{\boldsymbol{v}}}_{t-1}$ | Eq. 3 |

Fitting the three models to one group member (F2) suggests that the original model outperforms the distance weighted model (Table S2). The comparison between the original model and the weighted ${\hat{\boldsymbol{v}}}_{cm}$ model suggest no large difference in model performance. Looking at the differences in estimated beta values we find that the original model and the weighted ${\hat{\boldsymbol{v}}}_{cm}$ model largely estimate similar effects, whereas the distance weighted model largely conforms to the group only model (i.e., only an effect of ${\hat{\boldsymbol{v}}}_{cm}$) (Table S3).

Table S2: Model comparison using WAIC. The difference is measured as the expected log posterior density between the original model and subsequent expansions on the model. Positive values indicate a preference for the original model, and standard errors of the difference are presented to help interpret the uncertainty around the difference.

| **Model** | **elpd_diff_WAIC_** | **se_WAIC_** |
| --- | --- | --- |
| Original | 0 | 0 |
| Weighted ${\hat{\boldsymbol{v}}}_{cm}$ | -3.9 | 2.5 |
| Distance weighted ${\hat{\boldsymbol{v}}}_{i,j}$ | 34.8 | 11.7 |

Table S3: Estimated beta coefficients from each model compared. Bold values indicate estimated beta coefficients with 99%CI not including zero.

| **Parameter** | **Original model** | **Weighted** ${\hat{\boldsymbol{v}}}_{\boldsymbol{cm}}$ | | **Distance weighted** ${\hat{\boldsymbol{v}}}_{\boldsymbol{i,j}}$ | |
| --- | --- | --- | --- | --- | --- |
| kappa | **1.3504** | **1.3535** | **1.3368** | |  |
| beta_M2 | **0.1537** | **0.139** | -0.1289 | |  |
| beta_F11 | **0.0651** | 0.0487 | 0.1104 | |  |
| beta_F2 | **0.0793** | **0.0685** | 0.1716 | |  |
| beta_F9 | 0.0124 | -0.0053 | -0.0239 | |  |
| beta_F4 | 0.0182 | 0.0109 | -0.1018 | |  |
| beta_F10 | -0.0122 | -0.0208 | 0.0264 | |  |
| beta_F8 | -0.0002 | -0.0144 | 0.0573 | |  |
| beta_F7 | 0.0094 | 0.0054 | 0.0138 | |  |
| beta_F6 | 0.0367 | 0.0403 | -0.0101 | |  |
| beta_F1 | **0.1211** | **0.1078** | 0.0329 | |  |
| beta_M1 | **0.2097** | **0.1896** | 0.0579 | |  |
| beta_F5 | 0.0389 | 0.0315 | -0.0531 | |  |
| beta_cm | **0.1289** | **0.3015** | **0.7515** | |  |

**References:**

1 Bonnell, T. R., Henzi, S. P., Barrett, L. 2016 Direction matching for sparse movement data sets: determining interaction rules in social groups. *Behavioral Ecology*. (10.1093/beheco/arw145)

2 Rivest, L.-P., Duchesne, T., Nicosia, A., Fortin, D. 2016 A general angular regression model for the analysis of data on animal movement in ecology. *Journal of the Royal Statistical Society: Series C (Applied Statistics)*. **65**, 445-463. (10.1111/rssc.12124)

3 Gelman, A., Pardoe, I. 2006 Bayesian measures of explained variance and pooling in multilevel (hierarchical) models. *Technometrics*. **48**, 241-251.
